# Personalized Predictive Model to Predict Subtask Success of Medication Adherence Technologies for Older Adults With Diverse Capabilities: Development and Internal Validation Study

**DOI:** 10.2196/84616

**Published:** 2026-04-08

**Authors:** Bincy Baby, Ghada Elba, Minzee Kim, SooMin Park, Imra Hudani, Rishabh Sharma, Halak Patel, Sidharth Bajaj, P K Patterson, Annette McKinnon, Sara J T Guilcher, Feng Chang, Linda Lee, Catherine Burns, Ryan Griffin, Joslin Goh, Tejal Patel

**Affiliations:** 1 School of Pharmacy, University of Waterloo, 10 Victoria Street South, Waterloo, ON, N2G 1C5, Canada, 1 519 888 4567 ext 21337; 2 Department of Statistics and Actuarial Science, University of Waterloo, Waterloo, ON, Canada; 3 Patient Advisor’s Network, Toronto, ON, Canada; 4 Leslie Dan Faculty of Pharmacy, University of Toronto, Toronto, ON, Canada; 5 Department of Family Medicine, McMaster University, Hamilton, ON, Canada; 6 Systems Design Engineering, Faculty of Engineering, University of Waterloo, Waterloo, ON, Canada; 7 National Research Council Canada, Ottawa, ON, Canada; 8 Schlegel-UW Research Institute for Aging, Waterloo, ON, Canada

**Keywords:** medication adherence, technology, older adults, personalized predictive modeling, usability

## Abstract

**Background:**

Older adults frequently experience cognitive, physical, sensory, motivational, and environmental barriers that affect medication management. Medication adherence technologies (MATs) can support adherence, but their usability varies widely depending on individual abilities and device features. Prior research has largely focused on overall adherence or user experience, providing limited insight into feature-level usability challenges.

**Objective:**

The aim of the study is to develop and internally validate a personalized predictive model to predict the success of MAT subtasks for older adults with diverse cognitive, physical, sensory, motivational, and environmental capabilities.

**Methods:**

A mixed methods approach was used, incorporating the assessment of impairments using various standardized questionnaires, measurement of usability metrics through cognitive walkthroughs, and one-on-one semistructured interviews. For this study, we used “subtasks” as the representative of features of the devices. A subtask is a discrete, individual action that forms part of a larger task, specifically designed to achieve a step in the overall process. Participants tested between 1 and 7 devices from a selection of 13 devices. The proportion of subtask success was taken as the outcome measure. Predictors included demographic, clinical, cognitive, physical, sensory, motivational, and environmental characteristics. Personalized predictive modeling using cosine similarity and generalized linear models were compared with nonpersonalized and naive models. Model performance was evaluated using mean square error (MSE) through cross-validation and held-out validation.

**Results:**

A total of 117 participants (mean age 74.6, SD 7.9 years) were recruited, including 96 participants for usability testing and 21 for the validation, all varying in cognitive, physical, sensory, motivational, and environmental abilities. Both personalized (*m*=0.25) and nonpersonalized models (*m*=1.0) outperformed naive predictions (*m*=1.21), demonstrating that subtask-level success can be predicted using routinely measurable demographic and functional characteristics. During cross-validation, personalized models achieved optimal performance at a matching proportion of *m*=0.25, with MSEs lower than those observed at higher matching levels, although differences compared with nonpersonalized models were not statistically significant (Self-Medication Assessment Tool [SMAT]: *P*=.50; Daily Living Tasks Dependent on Vision [DLTV]: *P*=.43). In the held-out validation cohort, personalized models achieved MSEs of 0.89 (SMAT-based) and 1.16 (DLTV-based) at m=0.20, whereas nonpersonalized models demonstrated better performance with MSEs of 0.726 (SMAT-based) and 0.815 (DLTV-based). Models incorporating performance-based vision measures (SMAT-based) consistently outperformed those using self-reported vision scores (DLTV-based) across both personalized and nonpersonalized settings.

**Conclusions:**

This study demonstrates the feasibility of predicting subtask success of MATs in older adults. While personalization showed limited added benefit in this dataset, the subtask-focused model provides clinically meaningful insights to support evidence-informed selection of medication technologies, reduce usability-related medication errors, and improve adherence outcomes.

## Introduction

As the global population ages, the proportion of older adults with chronic health conditions is increasing significantly [1]. According to projections by Statistics Canada, approximately 25% of Canadian people will be older than 65 years of age by 2036, highlighting the importance of creating support systems that allow these individuals to live independently and maintain their quality of life [2]. Chronic health conditions, including heart disease, diabetes, arthritis, and other ailments, are more prevalent in this age group, presenting various challenges that impair their ability to manage daily tasks, including the crucial task of medication management [1]. Complex medication regimens and polypharmacy, a common practice due to multimorbid conditions, introduce additional challenges that often lead to various medication-related problems, such as medication errors, potentially inappropriate medications, adverse drug reactions, and nonadherence, subsequently preventing the achievement of therapeutic outcomes [3-5].

Medication management capacity (MMC) is defined as the “cognitive and functional ability to comply with a medication regimen, when it is the individual’s wish or desire to follow a medication regimen as prescribed,” and is recognized as an important aspect of adherence [6]. In terms of MMC, deviations from adherence result from a lack of ability and are unintentional [6]. Various challenges associated with aging and chronic conditions can affect medication management, including cognitive, physical, sensory, motivational, and environmental barriers [6-9]. Cognitive decline, often exacerbated by chronic illnesses or age-related conditions such as dementia, presents challenges with memory, spatial orientation, and processing speed, which make it difficult for older adults to follow medication schedules accurately, increasing the possibility of errors and adverse events [9-12]. Physical impairments, such as reduced grip strength, fine motor skills, and hand dexterity, further compromise the self-management of medications. For instance, individuals with rheumatoid arthritis often struggle to open medication packaging due to reduced hand function, which can lead to missed doses and unintentional nonadherence [13,14]. Sensory impairments, including decreased visual acuity and hearing, also hinder medication adherence, as patients may be unable to read labels or hear auditory reminders [9,15-17]. Motivational factors, such as confidence in managing medications and understanding the benefits of adherence, play an important role as well [18-20]. Additionally, environmental factors like inadequate lighting at home, cluttered spaces, and a lack of support from caregivers or family can further complicate effective medication management [21-23].

Addressing these barriers is essential, as medication nonadherence can lead to increased health care costs, hospitalizations, and even preventable deaths [7,8,24,25]. Various studies have explored potential solutions through the development and implementation of assistive technologies to support older adults in managing their medications independently [26-29]. Medication adherence technologies (MATs), such as electronic pill dispensers, ingestible sensors, blister pack systems, and smart medication bottles, have emerged as aids, especially for older adults facing cognitive or physical impairments [26-29]. Research indicates that these technologies could improve adherence by providing alerts, reminders, and dosage tracking to simplify the medication-taking process [30,31]. A mobile health–based medication adherence measurement system using electronic blisters in 53 older adults with increased cardiovascular risk significantly improved adherence to diabetes medication (*P*=.04) during the monitoring phase. Similarly, another 6-month trial in primary care patients with moderate to severe asthma found that inhaler reminders and feedback significantly improved adherence (73% vs 46%; *P*<.001). However, how easy MATs are to use and how they appear to the user vary depending on individual user characteristics, impairments, and specific device features [32-36]. For example, individuals who are visually impaired may struggle with devices that rely solely on visual cues, and older adult users may have difficulty opening devices that require a lock and key or are too tight to open due to limited hand dexterity, highlighting the importance of selecting technologies with accessible features that match individual abilities.

Usability focuses on the ease, effectiveness, and efficiency with which users can complete tasks, while user experience (UX) encompasses the overall emotional and psychological interaction with a device, including satisfaction and engagement [37-39]. Both are critical for creating successful technologies, especially for older adults, whose cognitive, sensory, and motor impairments require designs that improve accessibility and independence [37-39]. Previous research on MATs has generally assessed UX without focusing on specific device features or the diverse challenges faced by users with different impairments [35]. Given that each older adult may have unique impairments and that MAT features vary widely, it is crucial to evaluate which specific features facilitate or hinder usability. Personalized predictive modeling (PPM) offers a promising approach by accounting for individual heterogeneity and enabling the prediction of feature-level success based on a user’s unique functional profile, thereby supporting more targeted and patient-centered MAT selection [40-42].

Therefore, the objective of this study is to develop and internally validate a personalized predictive model to predict the success of MAT subtasks for older adults with diverse cognitive, physical, sensory, motivational, and environmental capabilities. For this study, we used “subtasks” as the representative of features of the devices. A subtask is a discrete, individual action that forms part of a larger task, specifically designed to achieve a step in the overall process. It involves precise activities, such as inserting a battery or setting a date, which are essential for the successful operation of a device. By developing a personalized predictive model to predict subtask-level success, this study seeks to guide the selection of more user-friendly MATs that meet the diverse needs of older adults. Ultimately, this tailored approach aims to reduce medication nonadherence, minimize health risks, and support the independence of older adults by ensuring that the technologies they use are accessible and aligned with their unique capabilities.

## Methods

This paper adheres to the TRIPOD (Transparent Reporting of a Multivariable Prediction Model for Individual Prognosis or Diagnosis) guidelines for the development of a multivariable prediction model [43]. The TRIPOD checklist was used to ensure complete and transparent reporting of all relevant items (Checklist 1).

### Study Design

This study used a prospective mixed methods research design conducted in 2 parts: usability testing and a validation part. Both phases followed the same overall study procedures. This paper focuses specifically on the development and validation of the personalized prediction model. Other outcome variables collected in the study, including additional performance and perception-based measures and qualitative findings from one-on-one interviews, are reported in separate papers.

### Eligibility Criteria

Participants were eligible to participate in the study if they were 60 years or older of age and were willing and able to provide informed consent. Participants were excluded from the study if they were unable to speak or read English, were younger than 60 years of age, or were unwilling or unable to provide informed consent. For participants who were unable to provide consent, ones with cognitive impairment, consent was obtained from their caregivers.

### Sample and Recruitment

Participants were recruited using purposive and snowball sampling methods to ensure a diverse and representative sample. Initially, we recruited participants and measured their age-related abilities. However, after reaching 50 participants, we encountered challenges in recruiting an adequate number of individuals with diverse impairments. To address this, we modified our recruitment strategy by incorporating screening questions to identify specific symptoms, such as memory problems, hand tremors, limited hand movements, low hand grip strength, low vision, difficulty differentiating colors, blurred vision, low hearing, hand paralysis, and lack of sensation in the hands. Additionally, we included questions assessing medication adherence, such as how often participants skipped their medication due to a lack of motivation or a busy schedule. These modifications allowed us to target participants with specific impairments.

### Sample Size

The sample size calculation for the usability study was guided by Green’s formula for regression analysis (n≥50+8*m*), where *m* represents the number of independent variables (physical, cognitive, vision, hearing, motivational, and environmental impairments) [44]. Based on this criterion, a minimum sample size of 98 participants was required. Recruitment efforts aimed to ensure that at least 5 participants with each type of impairment tested each of the 13 selected devices, providing a comprehensive and balanced representation across diverse user needs. Recruitment and data collection took place from June 2023 to June 2024.

For internal validation, an increase in sample size by 30% was estimated to be adequate, corresponding to a target of 29 participants. Recruitment and data collection for the validation study took place from May 2025 to August 2025 [45,46].

### Study Procedure

An overview of the study procedures followed for the usability and validation phases is provided below. The study procedures remained exactly the same for both phases, with the exception that one-on-one interviews were not conducted during the validation phase.

#### Step 1: Measurement of Barriers to MMC

The first step involved assessing various barriers to medication self-management, including physical, cognitive, vision, hearing, motivational, and environmental factors. The following tools were used to determine these abilities:

- Self-Medication Assessment Tool (SMAT): Evaluated physical, cognitive, and vision barriers [47,48].
- Daily Living Tasks Dependent on Vision (DLTV) questionnaire: Focused on vision-specific barriers [49,50].
- Whisper test: Measured hearing impairment, categorizing it as 100% impairment if both ears failed, 50% if 1 ear failed, and no impairment if both ears passed [51,52].
- Self-Efficacy for Appropriate Medication Use Scale (SEAMS): Assessed motivational barriers related to confidence in adhering to medication regimens [20,53-55].
- Martin and Park Environmental Demands (MPED) questionnaire: Evaluated environmental challenges that could impact medication management [21].

#### Step 2: Usability Testing

##### Overview

After assessing barriers, participants were assigned MATs and performed tasks using these devices following the cognitive walkthrough approach. Two evaluators observed participants’ interactions with the devices, systematically documenting task and subtask outcomes, including success, failure, errors, and time taken. The evaluators then reached a consensus regarding the participants’ performance on each subtask to ensure consistency and reliability of the observations. More details on the cognitive walkthrough, the devices tested, and the mock medication regimen used in this study are provided below.

##### Cognitive Walkthrough

The cognitive walkthrough method evaluates how new users interact with a device by simulating their initial experience of exploring its functionality [37-39]. This task-based approach focuses on assessing ease of use and learnability, with the goal of identifying usability challenges that may impede task completion [37-39]. Participants were given a set of predefined tasks outlined in a participant information sheet, designed to evaluate various features and functions of the devices. Tasks ranged from simple actions, such as inserting batteries and unlocking devices, to more complex activities, such as setting alarms, filling medication trays, and retrieving medication after an alert. Each task consisted of smaller, discrete actions called subtasks. For example, in the case of inserting batteries, the subtasks included opening the battery compartment, correctly positioning the batteries, closing the compartment, and ensuring the device was powered on. Across the 13 devices evaluated, 32 subtasks were identified and generalized to ensure broad applicability across devices. The list of subtasks tested in this study is given in Table 1.

**Table 1.** Subtasks for using various devices.

Code	Subtask
A1	Locate the battery or cartridge compartment or medication cavity
A2	Put or insert the battery correctly
A3	Lift or close the battery compartment door
A4	Slide in or out the battery compartment door
A5	Slide a tab or button
A6	Check or ensure or verify the device is on or the lock is placed in position or follow instructions or ensure the indicator light flashes for 3 seconds
B1	Flip device
B2	Insert key and rotate
B3	Press and rotate the lid
B4	Open the lid by lifting
C1	Press and hold a button on a device
C2	Press a button on a device
D1	Open the pill box or compartment by rotating the lid
D2	Open the pill box or compartment or tray or door by sliding
D3	Pick up the correct pillbox or pill organizer or open correct compartment
D4	Insert or fill or place medication in the compartment or pillbox or pill organizer
D5	Close lid
D6	Put stickers on pillbox dividers
G1	Remove the medication
G2	Grab or hold the device
G3	Place hand over the open slot
H1	Rotate the carousel 3 days from today’s date
I1	Locate and touch on an icon or button on an app or screen
I2	Enter or type any data in an app or screen
I3	Scroll the screen options
M1	Align and insert the cartridge into the designated slot
P1	Tear the package
R1	Rotate retaining clips at each end of the device in an open or close position
R2	Align connectors to one another and gently push the card into the device
T1	Pierce cavity barrier
T2	Pinch number printed on the card and pull out
U1	Pull the blister packs away from the device

For each device tested, both a participant information sheet and an evaluation sheet were prepared. The information sheet provided instructions for the tasks participants needed to complete, while the evaluation sheet detailed each task and its associated subtasks. The evaluation sheet also recorded key metrics such as time taken for each task, whether each subtask was completed successfully without assistance, with assistance, or failed, and the presence or absence of errors during each subtask.

##### Device Tested

In this study, 3 smart and 10 electronic MATs were tested for usability and UX. Electronic MATs often include features like multiple compartments and alarms to remind patients to take their medications. In contrast, smart MATs incorporate advanced connectivity features, such as Bluetooth and Wi-Fi, enabling real-time tracking, remote monitoring, and instant feedback between patients, caregivers, and health care providers. These devices frequently include additional capabilities such as audio-visual reminders, adherence data logging, and report generation. The devices to be tested were selected to represent a wide diversity of features, based on a feature-based classification system developed for MATs [56]. Specifically, the selection aimed to include devices with variation across key dimensions of the classification system, including physical features, display, connectivity, system alert, data collection and management, operations, and integration. Devices were purposively chosen to capture differences in compartment design, dispensing and access mechanisms, alert modalities, portability, and levels of automation and connectivity. All selected devices were commercially available and commonly used for medication management. This approach ensured that the sample of MATs reflected a broad range of characteristics and functionalities relevant to older adults and supported a comprehensive testing of usability across diverse MATs. A list of the tested devices is provided in Multimedia Appendix 1. Each participant tested between 1 and 7 devices, with some participants requiring 2 visits to complete their testing. Initially, devices were assigned by the researchers without following specific methods or procedures. However, after testing 50 participants, the assignment strategy was revised to ensure at least 5 participants with each type of impairment tested each device, aiming for balanced representation across 6 types of impairments and devices. No formal training was provided to participants, who relied solely on the instructional materials included with the devices, simulating real-world conditions where users independently learn to use such devices.

##### Mock Medication Regimen

A simulated medication regimen was designed for this study to replicate the complexity of real-life medication schedules often managed by older adults. Developed in-house, this mock regimen aimed to assess the usability of technologies by mimicking the organization of medications in everyday of MATs while ensuring safety by substituting actual medications with placebo tablets, placebo capsules, and candy.

The mock regimen included placebo equivalents for common medications used to manage chronic conditions, structured as follows:

- Warfarin: 2 mg once daily on Monday, Wednesday, and Friday, and 3 mg once daily on Tuesday, Thursday, Saturday, and Sunday.
- Pantoprazole: 20 mg taken twice daily.
- Phenytoin: 100 mg, with 1 capsule in the morning and 2 capsules in the evening.
- Propranolol: 20 mg, with half a tablet once daily for the first 2 days, followed by 1 full tablet daily.

### Step 3: Qualitative One-on-One Interview

Following usability testing, participants engaged in one-on-one interviews to provide feedback on the devices they tested. These interviews explored their experiences, challenges, and perceptions of each device’s usability and features, offering qualitative insights to complement the quantitative data.

### Outcome Measure—Proportion of Subtask Success

The proportion of subtask success is a measure that shows how often participants successfully completed a specific subtask when using a device. This was calculated using the equation:


(1) $$\begin{array}{rl} Proportion\;of\;subtask\;success & =\frac{\begin{array}{r} number\;of\;times\;present\;in\;device\;use\\ -number\;of\;times\;participants\;failed \end{array}}{\begin{array}{r} number\;of\;times\;subtask\;present\\ in\;device\;use \end{array}} \end{array}$$

### Data Collection and Statistical Analysis

#### Data Management and Storage

All collected data from this study were securely managed and organized using the REDCap (Research Electronic Data Capture) platform (version 15.1.2; Vanderbilt University). This included participant demographics, outputs from the tools used to measure barriers to MMC, and the detailed data recorded in the evaluation sheets for each device tested. R Studio (version 4.4.1; Posit Software, PBC) was used to conduct data analysis.

#### Data Analysis Using PPM

The data analysis methodology used PPM to predict the success rates of subtasks for participants based on their unique characteristics. Unlike traditional modeling, where we use large and heterogeneous data to train the model, with personalized prediction modeling, we identify and analyze the data that are similar to the new participant of interest, whose outcome is to be predicted. By training the model on the subset of the data containing only the participants that are similar to the participant of interest, we aim to provide a customized prediction with improved predictive performance [57]. In other words, a new model is fit for each participant to predict their proportion of success.

There are a few key details of interest when implementing the model with PPM. The first is quantifying how similar one participant is to another participant, and the second is identifying the best subpopulation proportion (denoted as *m*) to train the unique model for each participant—in other words, how many similar participants do we use to train a predictive model for the participant of interest.

Our approach to measuring similarity is using the cosine similarity metric (CSM). CSM is intuitive to interpret, standardizes the similarity between −1 and 1, and can handle mixed data types that contain both categorical and continuous predictors. The data for each participant, such as their demographic and impairment information, are represented as a Euclidean vector of predictors in a multidimensional feature space, and CSM is used to measure the angle between the vectors for 2 participants. CSM quantifies the similarity between 2 participants as a cosine of an angle with values between −1 and 1, where −1 represents minimum similarity, and 1 represents maximum similarity [53]. For example, if CSM between participant A and B is −0.1, and CSM between participant A and C is 0.6, then C is more similar to A than B is to A.

With PPM, we train a model on a personalized dataset that contains only the most similar participants to the participant of interest. Then, it is crucial to determine what percentage of the most similar participants from the available data we will use to train the model (denoted as *m*). We find the optimal subproportion of the available data by tuning for this parameter using k-fold cross-validation (CV). The steps are as follows, adapted from previous literature on PPM [57]:

1. Randomly split the available data into 9 folds. The number of folds is 9 to allow the 117 participants to be distributed evenly along all the folds. One of the folds is the test data, and the rest of the data will be the training data.
2. For participant *j* in the test data, calculate CSM between participant j and every participant in the training data. Fit a generalized linear model (GLM) with a binomial logistic framework using the top *m×*100% of the most similar participants in the training data, and calculate the predicted proportion of subtask success for participant *j* using this model. Predictors in this model include demographic and clinical variables such as age, sex, number of medications, cognitive and physical scores, vision metrics (SMAT and DLTV scores), SEAMS scores, and hearing impairment.
3. Repeat step 2 for every participant in the test data. In other words, we are fitting a unique GLM with a logistic model using a tailored dataset containing only the top *m×*100% of the most similar participants from the training data for each participant in the testing data. Then, calculate the mean square error (MSE) to get the predictive performance for this fold. Predictors for each GLM included demographic and clinical variables such as age, sex, number of medications, cognitive and physical scores, vision metrics (SMAT and DLTV scores), SEAMS scores, and hearing impairment. Two models per participant were compared, one using SMAT vision scores and the other using DLTV vision scores, and success probabilities for subtasks were estimated based on model coefficients.
4. Repeat steps 2‐3 such that each fold becomes the test data, and the rest of the available data becomes the training data. Average over the MSEs calculated to get the predictive performance of the model for a specific *m* value.
5. Repeat steps 1‐4 for values of *m* ranging from 0.20 to 1.00, in increments of 0.05, to examine the effects of different matching percentages. Smaller subproportions (sub 0.2) were not explored because the resulting dataset would not be heterogeneous enough across all the features to fit the regression model. Note that as *m* increases, we have larger data to train the predictive model, but we also introduce more noise in the data as a trade-off. We denote *m* that gives the smallest MSE value as the optimum subpopulation proportion. When *m*=1, we are using a global (or nonpersonalized) model where we use the exact same data to fit the GLM model for each participant, because we are using all available dataset.

The above CV study can be used to tune for the optimal matching percentage *m* on the combined dataset of 117 participants. The recommended matching subproportion can be used when predicting the proportion of success at a subtask of a new test participant. However, this simulation cannot be used to estimate the generalization performance on unseen data since all of the data appear in at least 1 of the training folds. This is typically why a separate test set is held out from the training process. This is the data that the models have not trained on; hence, the model’s performance on this data is a good estimate of the model’s accuracy on unseen data. The dataset of 117 participants is split into the 96 participants collected up to 2024, which would be the training dataset, and the 21 participants collected in 2025, which would be the test data. This would give us an estimate of the performance of PPMs on unseen data and also provide us with benchmarks to outperform for newer models.

In both the above simulations (CV and model validation), model performance is quantified by the squared error of the model in predicting the number of successful attempts at a subtask. This evaluation metric allows us to view the problem in the lens of regression rather than as a classification task where area under the curve or *F*_1_-scores would be used. The choice is made because it is challenging to define the threshold for success at a subtask since multiple attempts are being made at subtasks, and an additional threshold hyperparameter that varies by subtask would be necessary.

#### The GLMs: Logistic Regression

Personalized subsets of participants were used to train GLMs for predicting subtask success rates. Two separate models were developed for each participant: (1) SMAT-based model using SMAT vision scores and (2) DLTV-based model using DLTV summary vision scores.

For each participant, a personalized GLM was fitted to their subset of training data identified via the CSM methodology. Predictors in the logistic regression model included demographics, clinical history, cognitive, physical, sensory, hearing, motivation, and environmental factor scores. Subtask-specific indicators were incorporated as covariates. Task count weights were applied to adjust for variations in subtask performance across participants.

The GLMs were structured as follows.

SMAT-based model:


(2) $$\begin{array}{rl} \text{logit}(Y_{i}) & =\beta_{0}+\beta_{1}\;{\text{sex}}_{i}+\beta_{2}\;{\text{mednum}}_{i}+\beta_{3}\;{\text{medhist}}_{i}\\ \;+\beta_{4}\;{\text{cog}}_{i}+\beta_{5}\;{\text{phy}}_{i}+\beta_{6}\;{\text{SMATvis}}_{i}\\ \;+\beta_{7}\;{\text{SEAMS}}_{i}+\beta_{8}\;{\text{MPEDbusy}}_{i}+\beta_{9}\;{\text{hear}}_{i}\\ \;+\beta_{10}\;{\text{subtask}}_{i} \end{array}$$

DLTV-based model:


(3) $$\begin{array}{rl} \text{logit}(Y_{i}) & =\beta_{0}+\beta_{1}\;{\text{sex}}_{i}+\beta_{2}\;{\text{mednum}}_{i}+\beta_{3}\;{\text{medhist}}_{i}\\ \;+\beta_{4}\;{\text{cog}}_{i}+\beta_{5}\;{\text{phy}}_{i}+\beta_{6}\;{\text{DLTVsumvis}}_{i}\\ \;+\beta_{7}\;{\text{SEAMS}}_{i}+\beta_{8}\;{\text{MPEDbusy}}_{i}+\beta_{9}\;{\text{hear}}_{i}\\ \;+\beta_{10}\;{\text{subtask}}_{i} \end{array}$$

where for a participant *i*, *Y_i_* is the proportion of success of a subtask, age*_i_* is the age, sex*_i_* is the sex, mednum*_i_* is the number of medications, medhist*_i_* is the number of medical history, cog*_i_* is the cognitive score, phy*_i_* is the physical score, SMATvis*_i_* is the SMAT vision score, DLTVsumvis*_i_* is the DLTV summary vision score, SEAMS*_i_* is the SEAMS score, MPEDbusy*_i_* is the MPED busyness score, MPEDroutine*_i_* is the MPED routine score, subtask*_i_* is a single subtask completed, and hear*_i_* is an indicator variable for hearing impairment.

### Ethical Considerations

This study was reviewed by and received approval from the University of Waterloo Office of Research Ethics (ORE##45203). All participants were informed of the study and provided consent before enrolling. Participant confidentiality was maintained by assigning unique participant ID numbers instead of names or identifiable information. All data were deidentified and securely stored. Participants received remuneration for their participation in the study. This study was conducted at multiple locations, including the University of Waterloo School of Pharmacy in Kitchener, Ontario, Canada; various Schlegel Villages across Ontario; and the KW Seniors Day Program in Kitchener, Ontario, Canada.

## Results

### Participant Demographics Characteristics

The study included a total of 117 participants (mean age SD 74.6, SD 7.9 years), including 96 participants for the usability testing and 21 participants for the validation, all with varying cognitive, physical, sensory, motivational, and environmental abilities.

**Figure 1.**
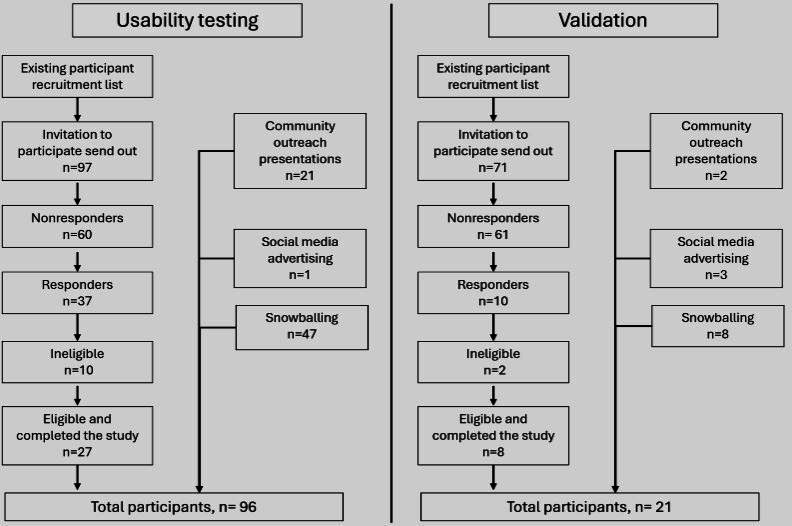
Participant recruitment and inclusion across the usability testing and validation.

Although we initially planned to recruit 98 participants for the usability testing and 29 participants for the validation, recruitment and inclusion challenges limited enrollment to 96 and 21 participants, respectively. One of our primary goals while recruiting was to ensure that each of the 13 devices was tested by at least 5 participants from each impairment group, which was achieved with the final usability sample and therefore considered sufficient for this component of the study. Additionally, considering that participants tested up to 7 devices, and that each device was treated as a distinct product with its own subtasks, the resulting dataset provided a sufficiently large number of observations. For validation, although the final sample size was smaller than planned, it allowed for an internal CV of the models. Further validation with larger samples is planned in future studies. Figure 1 presents a flow diagram summarizing participant recruitment and inclusion across the usability testing and validation. As the study involved a single assessment session (with a small number of participants completing 2 sessions), there were no participant attribution or dropouts during the study.

### Barrier Characteristics of Participants

Among the various barriers identified in the usability study, cognitive barriers, defined as low or moderate cognitive scores, were present in 21.87% (21/96) of participants, as determined using the SMAT. Physical barriers, defined as low or moderate to low physical scores, were observed in 38.54% (37/96) of participants. Vision barriers were also notable, with 23.95% (23/96) scoring low or moderate to low on the SMAT vision scale, and 38.54% (37/96) reporting impairments in DLTV, as assessed using the DLTV. Hearing barriers, evaluated with the whisper test, were present in 57.29% (55/96) of participants. Additionally, motivation and environmental challenges were reported by 35.41% (34/96) of the group (Table 2).

**Table 2.** Demographic, clinical, and barrier characteristics of study participants.

Variable	Usability sample (n=96)	Additional validation sample (n=21)
Age (years)		
Mean (SD)	75.11 (7.70)	73.42 (8.56)
Range	61‐95	60‐84
Sex, n (%)		
Male	36 (37.50)	14 (66.67)
Female	60(62.50)	7 (33.33)
Medication use (yes), n (%)	86 (89.58)	20 (95.24)
Level of education, n (%)		
Masters or doctoral or professional degree	26 (27.08)	6 (28.57)
Bachelor’s degree	27 (28.13)	9 (42.86)
Nonuniversity diploma	17 (17.70)	3 (14.28)
Trade certificate or diploma	1 (1.04)	1 (4.76)
High school	24 (25)	2 (9.52)
Below high school	1 (1.04)	0 (0)
Place of residence, n (%)		
Home	83 (86.46)	19 (90.48)
Retirement home	13 (13.54)	2 (9.52)
Self-reported medical history, n (%)		
Cardiovascular	56 (58.33)	0 (0)
Metabolic and endocrine	37 (38.54)	1 (4.76)
Musculoskeletal	26 (27.08)	11 (52.38)
Eye-related conditions	18 (18.75)	0 (0)
Oral and gastrointestinal	16 (16.67)	0 (0)
Neurological	12 (12.50)	3 (14.28)
Respiratory	10 (10.42)	0 (0)
Mental health	8 (8.33)	2 (9.52)
Renal and urogenital	7 (7.29)	2 (9.52)
Cancer and neoplasms	5 (5.21)	3 (14.28)
Stroke	5 (5.21)	4 (19.05)
None	5 (5.21)	4 (19.05)
Hematological diseases	4 (4.17)	0 (0)
Congenital disorders	2 (2.08)	3 (14.28)
Inflammatory and immune system	2 (2.08)	0 (0)
Injuries and accidents	2 (2.08)	1 (4.76)
Ear-related conditions	1 (1.04)	1 (4.76)
Infection	1 (1.04)	0 (0)
Reproductive health	1 (1.04)	3 (14.28)
Skin	1 (1.04)	4 (19.04)
Other	1 (1.04)	0 (0)
Medication management strategies, n (%)		
Pillbox	43 (44.79)	7 (33.33)
Alarm beeper	11 (11.46)	2 (9.52)
Blister pack	10 (10.42)	4 (19.05)
Someone else reminds me	8 (8.33)	1 (4.76)
Others	3 (3.12)	6 (28.57)
Medication calendar	2 (2.08)	1 (4.76)
Barriers		
Cognitive barrier—Self-Medication Assessment Tool, n (%)		
High cognitive scores (90% or greater)	70 (72.92)	14 (66.67)
Relatively high cognitive score (80% or greater)	5 (5.21)	1 (4.76)
Moderate cognitive score (70% to 80%)	7 (7.29)	2 (9.52)
Low cognitive score (approximately 69% or less)	14 (14.58)	4 (19.05)
Physical barrier—Self-Medication Assessment Tool, n (%)		
High physical scores (90% or greater)	59 (61.46)	15 (71.43)
Moderate to low physical score (76% to 84%)	11 (11.46)	3 (14.28)
Low physical score (approximately 75% or less)	26 (27.08)	3 (14.28)
Vision barrier—Self-Medication Assessment Tool, n (%)		
High vision scores (90% or greater)	73 (76.04)	18 (85.71)
Moderate to low vision score (76% to 84%)	4 (4.17)	1 (4.76)
Low vision score (approximately 75% or less)	19 (19.79)	2 (9.52)
Vision barrier—Daily Living Tasks Dependent on Vision^a^, n (%)		
Impairment present	37 (38.54)	10 (47.62)
Hearing barrier—whisper test, n (%)		
Impairment present (both ears)	55 (57.29)	12 (57.14)
Motivation barrier—Self-Efficacy for Appropriate Medication Use Scale^b^, n (%)	34 (35.42)	3 (14.28)
Environmental barrier—Martin and Park Environmental Demands^c^ questionnaire, n (%)	34 (35.42)	5 (23.81)

a Total score <79: vision barrier present.

b Total score <40 low self-efficacy: motivational barrier present.

c Busyness subscale score ≥15—greater busyness, routine subscale score <16—less routine: environmental barrier present.

In the additional validation sample, similar patterns were observed. Cognitive barriers were present in 28.57% (6/21) of participants, physical barriers in 28.57% (6/21), and vision barriers in 14.28% (3/21) based on SMAT scores. Impairments in vision-dependent daily living tasks were reported by 47.62% (10/21) of participants, and hearing barriers were present in 57.14% (12/21). Motivational and environmental barriers were less prevalent in the validation sample, reported by 14.28% (3/21) and 23.81% (5/21) of participants, respectively (Table 2).

Table 2 presents the demographic, clinical, and barrier-related characteristics of the study participants.

### PPM Outcomes

**Figure 2.**
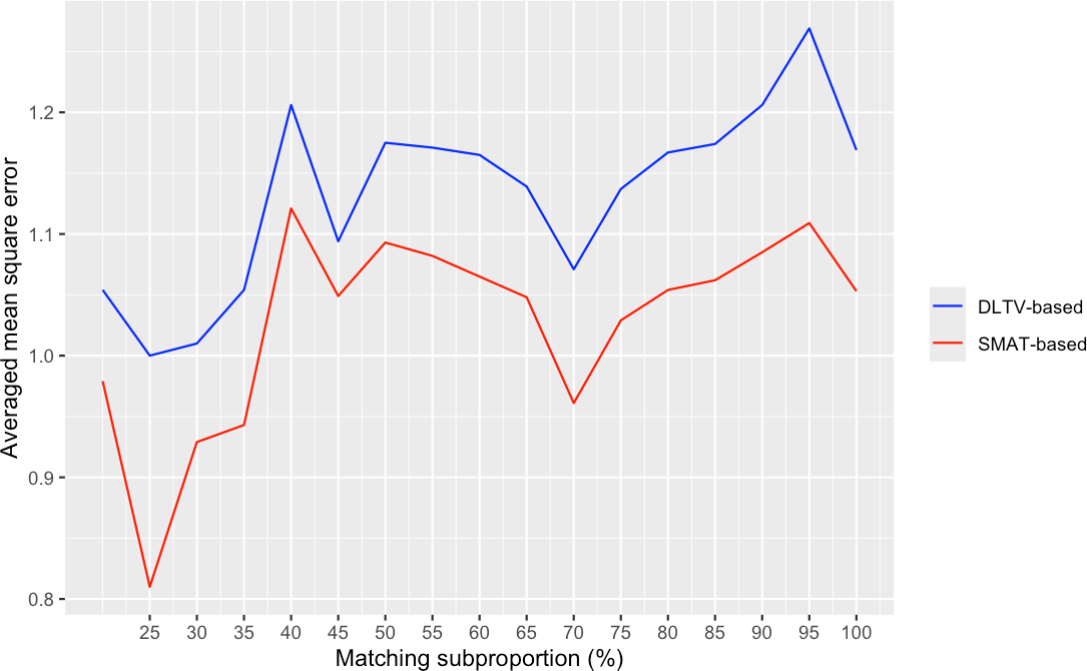
Mean square error of the DLTV-based and SMAT-based models averaged over all k-iterations of the cross-validation study as a function of the matching percentage *m*. DLTV: Daily Living Tasks Dependent on Vision; SMAT: Self-Medication Assessment Tool.

We begin by illustrating our choice of the tuning parameters and how they impact each of the PPMs. Figure 2 indicates that *m*=0.25 is the optimal matching percentage in this scenario for both the SMAT-based and DLTV-based models. Note that the averaged MSE decreases from *m*=0.2 to *m*=0.25 in Figure 2. Therefore, it is not necessary to modify the matching strategy to accommodate PPMs to be trained on the dataset with lower matching percentages (less than 20%) since the optimal matching subproportion as illustrated by Figure 2 is greater than *m*=0.2. The fairly volatile nature of the trends in Figure 2 suggests that tuning the matching parameter is beneficial, as there is a large difference between the MSE at *m*=0.25 compared to the MSE at *m*=0.95, where performance is even worse than not using any personalization at all (*m*=1.0).

Paired 2-sided Wilcoxon signed rank tests were conducted to determine if there is a statistically significant difference in the median MSE values (along the k-folds) between the personalized models at *m*=0.25 and their nonpersonalized counterparts (*m*=1.0). These tests (DLTV: *P*=.43; SMAT: *P*=.50) do not show statistical significance. Hence, even though 25% matching is optimal in Figure 2, the MSE populations may not be statistically different enough to necessitate tuning for the matching parameter.

If the datasets become large enough that matching each new participant to *m×*100% of the training dataset and refitting a new model becomes a computational bottleneck, skipping the personalization step should not statistically degrade model performance. However, when computation is not a constraint and PPMs can be used, the optimal matching percentage with respect to the current data is *m*=0.25.

### Model Validation

The CV procedure as described in the previous section was carried out on the 96 participants but with 8 folds instead of 9 folds, and it was found that the optimal matching percentage was 0.2 for both the DLTV-based and SMAT-based models. On the test set, the SMAT-based model with a matching parameter of 0.20 achieves an MSE of 0.89, and the DLTV-based model with a matching parameter of 0.2 achieves an MSE of 1.16. In other words, the SMAT-based model is off by 0.94 subtasks when predicting the correct number of subtasks a participant has successfully completed, while the DLTV-based model with a matching parameter of 0.20 is off by 1.1 subtasks. Note that these results do not reflect the best possible performance of the models on the test set because the matching parameter, while optimal for the training set, is not necessarily optimal for the test set. The nonpersonalized models (*m*=1.0) perform much better with an MSE of 0.726 and 0.815 for the SMAT and DLTV-based models, respectively. This does raise the question of whether the optimal matching percentage *m* transfers over to unseen data that might have a different distribution compared to the training data.

To contextualize these results, note that the average proportion of success in the training data collected in 2024 is 0.87. Consider a naive model that always predicts a fixed proportion of success regardless of the participant’s features. Due to the left-skewed nature of the success variable, the naive model predicting a success rate of 100% would do much better than a naive model that predicts a fixed success rate of 0%. To be more precise, the naive model that minimizes MSE on a dataset predicts a constant probability equal to the inner product of the success counts and the corresponding number of attempts across subtasks normalized by the squared l^2-norm of the number of attempts at a subtask. Since the test data are unseen, the naive model can instead compute this ratio on the training set and apply it on unseen data. Numerically, this is a prediction of 0.84 resulting in an MSE of 1.21. The optimal naive model is on average 1.1 subtasks off in predicting the correct number of subtasks.

Overall, the SMAT-based model with *m*=0.2 is on average more accurate by 0.07 subtasks in prediction, while the DLVT-based model with *m*=0.2 performs similarly to the naive baseline. In contrast, in the nonpersonalized setting (*m*=1.0), the SMAT-based model improves accuracy by 0.25 subtasks, and the DLVT-based model by 0.2 subtasks relative to the naive model. The naive model’s MSE of approximately 1.1 subtasks serves as a useful benchmark for future model development and validation studies.

It is possible that the training-testing split is suboptimal if participants recruited during the first phase of data collection (2023‐2024) systematically differ from those recruited during the second phase (2025). To assess this, statistical tests were conducted on all participant features used in the model to evaluate differences between the 2 cohorts. After adjusting for multiple-comparison error, no statistically significant differences were observed for any feature. This finding supports the chosen training-testing split. However, given the unequal and relatively small sample sizes of the 2 cohorts (96 vs 21), these tests may have been underpowered. Consequently, a random partition of the combined dataset (n=117) may have provided a more statistically robust assessment of both the benefits of personalization and the performance of naive models.

### Model Specifications

Unlike conventional prediction models that estimate a single set of regression coefficients applicable to all individuals, PPM generates individualized models tailored to each participant. Specifically, each participant’s model is trained on a personalized dataset comprising observations most similar to that participant. Consequently, for the 117 participants included in this study, 117 distinct logistic regression models were estimated, each with its own set of regression coefficients and intercept. Because model parameters are individualized by design, there is no single, global set of regression coefficients, odds ratios, or CIs that can be meaningfully reported. Instead, model specification is defined procedurally rather than through fixed parameter estimates.

To generate predictions for a new individual, we first apply steps 1‐5 of the PPM procedure (see Data Analysis Using PPM section) to the full available dataset to determine the optimal subpopulation proportion, *m*. This proportion defines the size of the personalized training set. We then identify the *m*×100% of observations most similar to the new individual according to the predefined similarity metric and fit a logistic regression model on this subset. The resulting personalized model can be used to estimate the probability of subtask success for that individual and to inform individualized recommendations.

## Discussion

### Principal Findings

This study was conducted to develop and internally validate a personalized predictive model to estimate subtask success of MATs among older adults with diverse cognitive, physical, sensory, motivational, and environmental abilities. Overall, the findings show that it is feasible to predict subtask success of MATs using measurable functional and demographic characteristics, and that such models can perform better than naive prediction models. These results demonstrate that individual differences meaningfully influence how older adults interact with MATs and that these differences can be quantified and modeled.

Research evaluating the usability of MATs for older adults, while considering their specific impairments, remains limited [28,35]. One study by Woo [58] examined usability through a small-scale device trial involving 2 older adult participants who tested a single device over a 2-day period. The study assessed user perceptions, adherence rates, and usability concerns through observations and interviews. In this study, the first participant was an 82-year-old female with mild cognitive impairment, arthritis, osteoporosis, and mobility challenges, requiring the use of a 4-wheeled walker. The second participant was an 84-year-old male with mild to moderate cognitive impairment, hypertension, and mobility impairments. While both achieved high adherence rates (100% and 90%, respectively), they encountered usability challenges. One participant struggled to grasp pills and had to tip the device over to retrieve medication, while the other faced difficulty closing pill compartments due to manual dexterity impairments. This underscores the importance of examining specific interaction failures related to device features and users’ functional abilities.

By focusing on subtasks rather than overall device success, this study addresses an important gap in MAT usability research. As we previously noted, subtasks represent features of the device, the specific actions required to use a device, such as pressing buttons, opening compartments, aligning parts, or reading visual indicators. Evaluating success at this level provides a more detailed and clinically meaningful understanding of usability than global device ratings, which may mask specific interaction challenges. This subtask-based approach allows for a clearer link between user abilities and the demands imposed by individual device features. The decision to model success at the subtask level is supported by assistive technology and human factors literature, which emphasizes that devices should be matched to users based on their functional limitations rather than by device type or overall complexity [59,60]. For example, individuals with mild hand weakness may succeed with simple, low-technology solutions, whereas those with more severe impairments may require devices that reduce physical or cognitive effort by automating certain actions [60]. MATs vary widely in the features, functionalities, effort, and understanding they require, and identifying subtask-level success makes it possible to identify which specific features could create barriers for particular users as well as which features are more suitable. Human factors research further shows that usability failures often occur at discrete interaction points, especially as device complexity increases [61-64]. By treating subtasks as representative device features, this study supports a more user-centered approach to MAT selection that recognizes assistive technologies as essential supports that must align closely with users’ abilities for successful use, acceptability, and adoption [61-65].

With respect to modeling strategy, both personalized and nonpersonalized GLMs improved prediction accuracy compared to a naive baseline that assumed uniform subtask success across users. This finding suggests that incorporating individual-level characteristics, such as cognitive function, physical ability, vision, hearing, motivation, and environmental factors, adds meaningful explanatory value when predicting how older adults interact with specific device subtasks. These results support the broader view that usability arises from the interaction between user characteristics and device features rather than by either factor alone. Although PPM is conceptually appealing, its advantages over nonpersonalized (global or broader) models were not statistically significant in this study. Personalized models showed some improvement during CV but did not significantly outperform nonpersonalized models overall, and in the held-out validation cohort, nonpersonalized models demonstrated better performance. Similar patterns have been reported in prior work. Ng et al [66] showed that personalized predictive models can outperform global models when trained on large, dense datasets with sufficient numbers of clinically similar cases, but also emphasized that reliable personalization depends on stable similarity estimates and adequate neighborhood size. When these conditions are not met, global models that leverage the full dataset may generalize more effectively. Evidence from outside health care further supports this interpretation; for example, in sports performance research, Ahsan et al [67] reported that while personalized modeling can offer targeted insights, broader predictive models are often more robust and easier to maintain when data are limited or heterogeneous. Together, these findings suggest that the lack of statistical significance between the models in this study likely reflects constraints related to sample size and population heterogeneity rather than a lack of conceptual value of personalized modeling itself.

The validation results highlight the importance of evaluating model performance beyond internal training. The stronger performance of nonpersonalized models in the validation cohort suggests that matching parameters optimized during training may not fully transfer to unseen data, particularly when sample sizes are small. Although no statistically significant differences were observed between recruitment phases, the smaller validation cohort may have limited the ability to detect subtle differences. These findings underscore the need for external validation in larger and more diverse populations before using PPM approaches in real-world clinical settings.

An additional important finding was that models incorporating SMAT-based (performance-based) vision measures consistently outperformed those using DLTV-based (self-reported) vision scores. This suggests that task-specific functional assessments may be more informative for predicting technology interaction than broader measures of daily living performance. However, at the same time, it is also important to consider the practical advantages of using task-based measures compared with self-reported measures [68].

From a clinical perspective, this modeling framework has the potential to support more informed MAT selection by predicting which device features an older adult is most likely to use successfully or struggle with. By estimating subtask-level success, the model can help clinicians, caregivers, and users anticipate whether specific device features are well matched to an individual’s cognitive, physical, sensory, and environmental abilities. This enables more informed decision-making when selecting MATs, particularly by avoiding devices with features that may be difficult or unsafe for a given user. Because medication management is a safety-critical activity, mismatches between user abilities and device demands can increase the risk of medication errors and nonadherence. Selecting technologies that align with users’ capabilities may reduce trial-and-error device adoption, minimize frustration, support correct device use, and ultimately could improve medication adherence, safety, and therapeutic outcomes.

### Strengths

This study has several strengths, including a diverse sample of 117 older adults with varying cognitive, physical, sensory, motivational, and environmental barriers. Given the exclusion of older adults with impairments in many studies, this inclusion improves the study’s relevance and applicability. Additionally, the study tested both electronic and smart MATs, allowing for a comprehensive comparison of their effectiveness. Another key strength is that it addresses multiple barriers affecting medication self-management, beyond just physical or cognitive impairments. The study’s use of validated assessment tools (eg, SMAT and whisper test) strengthens its methodological quality, ensuring reliable and meaningful findings. Finally, the subtask-based success approach provided detailed, feature-level insights into usability, supporting more clinically meaningful interpretations of device-user interactions.

### Limitations

Despite its strengths, the study has some limitations. Testing environments may not fully replicate real-world conditions, potentially affecting the generalizability of findings. Additionally, while the sample is diverse, it may not fully capture all cultural and socioeconomic factors influencing MAT use. The variability in participants’ technological literacy and health status could also introduce biases, impacting the study’s applicability to the broader older adult population. A further limitation is related to sample size planning and model complexity. The initial sample size estimation for the usability phase was guided by Green’s formula based on 6 primary impairment domains. However, the final predictive models included a larger set of predictors, including demographic, medication-related variables, multiple functional scores, environmental factors, hearing scores, and subtask indicators. While each personalized model was trained on a subset of similar participants (approximately 20%‐25% of the training sample), the ratio of observations to predictors may still be limited, increasing the risk of overfitting and reducing coefficient stability. This constraint likely contributed to the modest performance gains observed for personalized models compared with nonpersonalized models and highlighted the need for larger datasets to fully support more complex personalized modeling approaches.

### Future Recommendations

Future research should address these limitations by conducting real-world studies in home and community settings and including a more representative demographic sample. Additionally, developing a standardized, comprehensive assessment tool for measuring all medication management barriers would improve consistency and applicability. Further studies could explore the longitudinal impacts of MATs on long-term adherence, hospitalization rates, and health care costs. Research should also focus on developing clinical decision-support tools that integrate usability metrics and patient-specific barriers, helping health care providers tailor recommendations. Examining the learnability and long-term engagement of older adults with these technologies is also important, as older adults may experience technology fatigue or changes in ability that affect long-term use. Finally, external validation of the predictive models in larger, independent, and more diverse populations is essential to assess generalizability and robustness. Future work should evaluate model performance across different health care systems, cultural contexts, and device types and examine whether personalized modeling approaches yield greater benefits as datasets grow, and similarity estimates become more stable. Such efforts can be beneficial for translating predictive usability modeling into routine clinical practice.

### Conclusions

This study developed and internally validated a personalized predictive model, demonstrating the feasibility of predicting subtask success of MATs among older adults using demographic, clinical, and functional characteristics. Both personalized and nonpersonalized models outperformed the naive model, highlighting the importance of individual differences in technology usability. Although personalization showed limited added benefit in this dataset, the subtask-focused approach offers clinically meaningful findings to support evidence-informed selection of medication technologies and thereby improving adherence outcomes.

## Supplementary material

10.2196/84616 Multimedia Appendix 1 List of medication adherence technologies tested.

10.2196/84616 Checklist 1 TRIPOD checklist.

## Abbreviations

CSM: cosine similarity metric
CV: cross-validation
DLTV: Daily Living Tasks Dependent on Vision
GLM: generalized linear model
MAT: medication adherence technology
MMC: medication management capacity
MPED: Martin and Park Environmental Demands
MSE: mean square error
PPM: personalized predictive modeling
REDCap: Research Electronic Data Capture
SEAMS: Self-Efficacy for Appropriate Medication Use Scale
SMAT: Self-Medication Assessment Tool
TRIPOD: Transparent Reporting of a Multivariable Prediction Model for Individual Prognosis or Diagnosis
UX: user experience
