# Supplementary material for: Personalized Predictive Model to Predict Subtask Success of Medication Adherence Technologies for Older Adults With Diverse Capabilities: Development and Internal Validation Study
Source: JMIR Aging. 2026 Apr 8;9:e84616. doi: 10.2196/84616 (PMC13060742; doi:10.2196/84616)
Supplement: Multimedia Appendix 1 [file aging-v9-e84616-s001.docx]

| 1. MedReady 1700 Automated Medication Dispenser |
| --- |
| 1. GMS Med-e-lert Automatic Pill Dispenser |
| 1. MedQ Smart PillBox |
| 1. MedGlider System 1 with Talking Reminder |
| 1. VitaCarry Advanced Pill Case |
| 1. e-pill Multi-Alarm Pocket XL |
| 1. 100-Hour Pill Reminder |
| 1. eNNOVEA Weekly Planner with Advanced Auto Reminder |
| 1. Pill Box with Digital instruction |
| 1. MedCentre System |
| 1. Spencer Automatic Pill Dispenser |
| 1. CpaX™ Connected Medication Adherence Packaging |
| 1. EllieGrid Smart Pill Organizer |

Predictive modeling to determine the usability of medication adherence technology among older adults
